# The influence of cognitive level on the guaranteed behavioral response of landless farmers in the context of rural revitalization–An empirical study based on partial least squares structural equation modeling

**DOI:** 10.3389/fpsyg.2022.967256

**Published:** 2022-11-22

**Authors:** Yangjie Lu, Hao Dong, Huanyuan Wang

**Affiliations:** ^1^China Shaanxi High Standard Farmland Construction Group Co., Ltd., Xi’an, China; ^2^Shaanxi Provincial Land Engineering Construction Group Co., Ltd., Xi’an, China

**Keywords:** cognitive hierarchy, landless farmers, guaranteed behavioral response, distributed cognitive theory, structural equation modeling

## Abstract

With the continuous acceleration of urbanization and agricultural modernization in China, the trend of concentration of rural land transfer is irreversible. For landless farmers, the absence of land guaranteed function inevitably gives rise to the substitution effect of other guaranteed methods. And the subjective preferences exhibited by farmers in making guaranteed behavior decisions can be quantitatively described as guaranteed behavioral responses (GBRs). Based on the analytical framework of distributed cognitive theory, this paper adopts the validated factor analysis method of structural equation modeling to quantitatively study the cognitive basis and behavioral responses of landless farmers’ guaranteed behavior by combining the survey data of rural households in typical rural areas of Wuhan urban area. The study shows that the GBRs of landless farmers are significantly influenced by the cognitive level. “Locality power,” “cultural power,” and “personal power” are the main, important, and effective cognitive levels that influence farmers’ GBRs, respectively. Policy-based protection occupies a dominant position in the rural social guaranteed system, savings-based protection still plays an important function in rural areas, and market-based protection has greater development potential.

## Introduction

China is currently in the stage of rapid industrialization and urbanization, and a large amount of agricultural land in good locations around cities has been expropriated. It is projected that the urbanization rate in China will exceed 75% in 2030, with an average annual increase of 1−2%, and the new urban population will be about 17 million per year, while the number of landless farmers will exceed 78 million (Bao and Peng, 2016), and landless farmers have become a huge interest group at this stage. In reality, local governments and developers often argue and contradict with farmers over land acquisition compensation and resettlement measures, and many farmers do not want their land to be expropriated, resulting in conflicts of wills and more serious consequences of violent conflicts.

Since the “globalization of land expansion” in 2000, conflicts about landless peasants have been happening continuously and have become more and more intense. Therefore, scholars have begun to focus on the conflict behavior of landless peasants, and have conducted many useful explorations from different perspectives. Bao et al. (2021) point out that in the past 10 years, the attitude of land-lost farmers toward land acquisition has changed greatly, from the initial compromise and acceptance to resistance and rights protection. He believes that China’s social transformation and urbanization process awaken the land-lost farmers’ awareness of land protection, and then affect land-lost farmers’ land acquisition conflict behavior (Li et al., 2020; Su et al., 2020). Liu et al. (2018) analyzed the formation mechanism of land-lost farmers’ right protection behavior in land expropriation from the micro level, and applied Logistic model to analyze the influencing factors of land-lost farmers’ right protection behavior. Mishra and Mishra (2017) believes that economic interest factors (per capita income level of family, Engel’s coefficient, compensation ratio of land expropriation loss) have a greater impact. Through the game analysis of the behaviors of local government, central government and peasant households in land expropriation conflicts, Cao and Zhang (2018) found that peasant households’ rights protection behavior depends on the cost of rights protection, compensation standard and land expropriation behavior of local governments. Bao et al. (2020) use the theoretical framework of social action to study the action strategies of land-lost farmers in the land expropriation environment, and believes that the changes of land-lost farmers’ rights protection methods and behavioral strategies at different stages are to maximize their own interests. Based on Korf’s scenario model and in combination with India’s macro-social and economic environment, Ghelichi et al. (2018) proposed the context-scenario model and applied it for the first time to study farmers’ participation in land acquisition conflicts in India, explored the influencing factors and action paths of farmers’ conflict behaviors, and concluded that the relationship between cadres and groups in rural society should be mainly managed. By studying the living conditions of peasants in Southeast Asia, Printsmann et al. (2022) proposed the concepts of “survival theory” and “moral economy,” arguing that when peasants’ survival morality and social justice are violated, they will have a strong will to resist and even resort to desperate measures. Other scholars, from the perspective of social problems in rural China, argue that there is a significant correlation between the occurrence of mass incidents in rural China and the rate of urbanization, and that the rent-seeking behavior of local governments and the lack of legitimate organizations representing farmers’ rights and interests have led landless farmers to resort to non-institutionalized violence to defend their rights. Through a review of the existing literature, it can be found that previous studies on landless farmers’ conflict behavior have mainly focused on the choice of conflict behavior, the context of behavior generation and causes, while not enough attention has been paid to the psychological behavior of individuals or groups in the process of land acquisition. In general, the literature on conflict willingness in China is scarce, and most of it remains in the qualitative research stage, and no scholars have conducted a comprehensive and systematic study on landless farmers’ conflict willingness.

The research methods are mostly limited to statistical analysis and quantitative analysis, and the research conclusion are not the same. On the basis of summarizing the existing literature, this paper aims to explore the cognitive logic of the guaranteed behavior decision-making of land-lost farmers under the current situation, and further study the cognitive basis behind the guaranteed behavior of farmers by combining the micro survey data of farmers in Xi ’an urban circle and the confirmatory factor analysis method of structural equation model. It also provides policy suggestions for improving risk management and guaranteed level of land-lost farmers and promoting rational improvement of rural social guaranteed system.

## Theoretical analysis and research hypotheses

**FIGURE 1:**
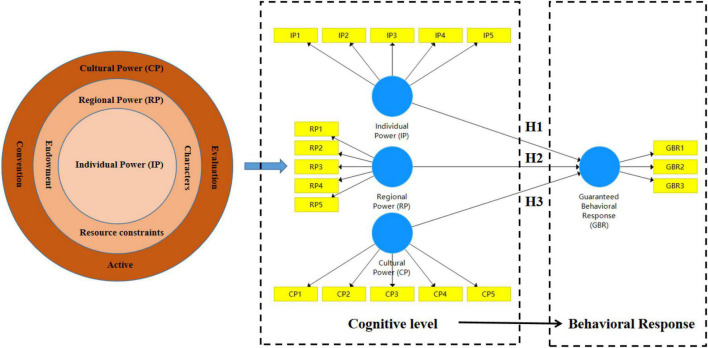
Research framework.

Psychology has a long history of research on individual cognitive activity, and Hatch’s research team pioneered the concentric circles model of distributed cognition (see Figure 1) back in 1993 (Rogers and Ellis, 1994), which posits that individual cognition is influenced by a combination of individual human, regional, and cultural power (Liu et al., 2008). Distributed Cognition Theory (DCT) was thus born. As a new perspective to observe the complete process of cognitive activities, distributed cognition no longer emphasizes the influence of individual characteristics on cognitive activities unilaterally, but takes the cognitive level of individuals’ processing of environmental information as the basic unit of research (Roessler et al., 2022). Relevant empirical studies have proved that DCT has strong explanatory power for individual cognitive activities in complex environments, and its analytical framework is applicable to the study of farmers’ behavior (Yakhlef, 2008).

The guaranteed behavioral response (GBR) studied in this paper is a customized abstract concept that connotes the subjective preferences that farmers exhibit when making guaranteed behavioral decisions (Lyver et al., 2019). According to the basic framework of distributed cognition (Belland, 2011), the cognitive environment of farmers can be subdivided into three cognitive levels: individual power (IP), regional power (RP), and cultural power (CP) for the specific problem of GBR (Lu et al., 2020), and there is a theoretical influence path of “cognitive level → behavioral response” (see Figure 1). Based on this, the hypothesis to be tested in this paper is as follows.

Individual power is the basis and core of distributed cognition, located in the innermost circle of the concentric circle model (Yen and Tsao, 2020), which emphasizes the characteristics and subjective motivation of the cognitive subject (Stiller and Schworm, 2019). The individual characteristics that may have significant influence include gender, age, social class status, and education level of farmers, while the effective manifestation of their subjective motivation is their part-time work ability (Jordakieva et al., 2020). Theoretically, the cognitive level of individuals is proportional to their growing experience, and their behavioral decision-making process will tend to be rational with the accumulation of experience.

**Hypothesis (H1).** IP is positively related to GBR.

Regional power is the key of distributed cognition, which is located in the middle layer of concentric circle model and emphasizes the interaction between cognitive subject and cognitive environment (Meyer et al., 2019; Valles-Colomer et al., 2019). When choosing the guaranteed mode, farmers will make behavioral decisions to a large extent depending on certain family roles, so the family is the main cognitive environment for individual farmers at this time (Meert et al., 2005). For this special group of land-lost farmers, the family environmental factors that may have a significant impact include family income, livelihood resilience, land transfer ratio, life attitude, and family happiness (Fang et al., 2016). The above indicators can measure the quality of family life of farmers to a certain extent, and theoretically farmers are more inclined to guarantee high-quality family life.

**Hypothesis (H2).** RP is positively related to GBR.

Cultural power is an abstract event that can have an indirect influence on specific cognitive activities (Gardner et al., 2000). It is located in the outermost layer of concentric circle model, and its influence on cognitive activities cannot be ignored (Jang and Kim, 2019). Farmers mainly live in traditional villages inhabited by acquaintances (Zhao and Zou, 2017), and their social relationship network is relatively simple. Their choice of guaranteed mode is mainly influenced by herd psychology, policy publicity, and policy satisfaction (Du et al., 2018). Theoretically, the direction of public opinion and public policy is largely the same, that is, to improve the level of rural social guaranteed and farmer household guaranteed is a basic consensus.

**Hypothesis (H3).** CP is positively related to GBR.

## Materials and methods

### Sampling and data collection

The questionnaire was answered by the most widely used questionnaire survey website “Questionnaire Star” in mainland China, and sampling was completed through the Ministry of Agriculture and Rural Affairs and the Shaanxi Provincial State-owned Assets Supervision and Administration Commission. Xi’an urban area is located in central Shaanxi Province, which is an important urban agglomeration in central China and a typical sample area for studying the “three rural issues.” The data used in this study were obtained from a survey conducted by the research team in May–June 2022 on farmland transfer households in a typical rural area of Xi’an city circle. The sample areas include but are not limited to Xi’an (Xizhangpo and Podi villages), Xianyang (Balizhuang, Baitu, and Yuanjia villages), Yan’an (Haojia and Shi’er villages), Yulin (Jinjiisha and Miaowan villages), Weinan (Beizhuang and Yohong villages), and Baoji (Jianhe and Wangjiazhuang villages). The sample sampling method was Accidental Sampling, and the survey was conducted by one-on-one semi-structured interviews with farmers. 450 questionnaires were distributed, of which 285 were valid, with a valid rate of 63.3%.

### Measures

The article contains four variables: IP, RP, CP, and GBR. The Likert 7-level scale is used to measure the above variables, and the measurement range is from “very dissatisfied” to “very satisfied” corresponding to the numbers “1” to “7.” Based on the above analysis and theoretical hypothesis, this paper designed a scale for measuring farmer household guaranteed behavior based on distributed cognitive theory (see Table 1). The scale contains 4 subject variables and 18 observation indicators. The influence paths among the 4 subject variables are shown in Figure 1, which constitutes the cognitive logic of farmer household guaranteed behavior. The 18 observation indicators are divided into 4 groups, and the specific meanings of 4 main variables are measured, respectively, so as to meet the quantitative research needs from “abstract concept” to “concrete reality.” On the basis of scale development and combined with the results of semi-structured interviews with farmers in the pre-survey stage, 18 questionnaire items were designed in this study, and the index values were represented in the form of ordered categorical variables of Likert scale. The specific scale development and questionnaire design results are shown in Table 2.

**TABLE 1:** Scale development and questionnaire design.

Characteristics	Samples	Percent (%)
Education level		
Illiterate	202	70.9
Primary school	15	5.3
Junior high school	12	4.2
High school	23	8.1
Bachelors	22	7.7
Master	9	3.1
PhD	2	0.7
Social identity		
Ordinary villagers	270	94.7
Village officials	15	5.3
Age		
<35 years	32	11.2
35–44 years	43	15.1
45–54 years	100	35.1
55–64 years	100	35.1
>65 years	10	3.5
Degree of part-time work		
Professional farmers	95	33.3
Professional–Part-time farmers	85	29.8
Part-time farmers	70	24.6
Part-time–Non-farmers	25	8.8
Non-farmers	10	3.5

**TABLE 2:** Descriptive characteristics of the sample.

Variable	Observation indicators	Measurement item	Sources
Individual power (IP)	IP1	Gender of survey respondents	Sirmon et al., 2007
	IP2	Age of survey respondents	
	IP3	Social class status of survey respondents	
	IP4	Education level of survey respondents	
	IP5	Extent of part-time employment of survey respondents	
Regional power (RP)	RP1	Average annual gross income of surveyed households in the past 3 years	Jehn, 1997
	RP2	How the employment status of survey respondents’ household labor force would change if existing livelihoods were unsustainable	
	RP3	Proportion of survey respondents’ household land transfer area	
	RP4	Survey respondents’ projections of household living conditions in the next 5 years	
	RP5	Survey respondents’ evaluation of family happiness at this stage	
Cultural power (CP)	CP1	The extent to which survey respondents accept “advice from acquaintances” when making decisions about safeguarding behaviors	Lee et al., 2010
	CP2	Receptiveness of survey respondents to “policy advocacy” when making decisions about safeguarding behaviors	
	CP3	Satisfaction level of survey respondents with agricultural subsidy policies	
	CP4	Satisfaction of survey respondents with rural pension insurance policies	
	CP5	Satisfaction of survey respondents with rural medical insurance policies	
Guaranteed behavioral response (GBR)	GBR1	Survey respondents’ agreement with “strict implementation of regular household savings plan	Cohen and Levinthal, 1990
	GBR2	Survey respondents’ recognition of “active participation in rural medical and pension insurance	
	GBR3	Survey respondents’ agreement with “buying insurance products provided by commercial organizations	

### Data analysis technique

Potential biases were considered in the survey, protocol design, and data analysis. Several approaches (e.g., direct contact by phone and assurance to share the results) were adopted to ensure the highest response rate and avoid a non-response bias (Frohlich and Westbrook, 2002). We used a partial least squares structural equation modeling (PLS-SEM) technique to analyze the data. This technique has been adopted because this process gives better results in the analysis of this type of exploratory study. This process can also analyze those data that are not normally distributed (Hair et al., 2012). This technique does not impose any sample restriction to conduct the survey. This process involves quantification of responses on a specific scale.

## Analysis result

### Measurement model assessment

The results showed that the measurement model satisfies all general requirements (see Table 3). First, all the standardized factor loadings of all the first-order and second-order constructs are above the minimum value of 0.808 (Fornell and Larcker, 1981). Second, the Cronbach’s alpha scores ranged between 0.707 and 0.934 while the composite reliability scores ranged between 0.866 and 0.930 which are above the recommended value of 0.70 indicating adequate construct validity. In addition, all the constructs have an AVE value above 0.50, suggesting that latent variables achieved convergent validity. Finally, this study follows three approaches to assess the discriminant validity, i.e, (1) Fornell–Larcker criterion, (2) cross loading, and (3) the Heterotrait–Monotrait ratio of correlations (HTMT).

**TABLE 3:** Reliability and validity.

Variable	Item	Convergent validity			Cronbach’s alpha	Multicollinearity
		Cross loadings	Composite reliability	AVE		VIF
Individual power (IP)	IP1	0.776	0.907	0662	0.873	2.033
	IP2	0.844				2.505
	IP3	0.846				2.247
	IP4	0.788				1.860
	IP5	0.811				1.795
Regional power (RP)	RP1	0.763	0.882	0.599	0.833	1.622
	RP2	0.812				1.808
	RP3	0.749				1.660
	RP4	0.783				1.781
	RP5	0.762				1.665
Cultural power (CP)	CP1	0.745	0.866	0.564	0.808	1.470
	CP2	0.771				1.613
	CP3	0.761				1.580
	CP4	0.770				1.736
	CP5	0.707				1.559
Guaranteed behavioral response (GBR)	GBR1	0.860	0.930	0.815	0.886	2.100
	GBR2	0.934				3.598
	GBR3	0.912				2.955

The correlation matrix in Table 4 shows that for each pair of constructs, the AVE square root of each construct is higher than the absolute value of their correlation (Fornell and Larcker, 1981). The results of cross loading show that all items are loaded higher on their respective constructs than on the other constructs and the cross-loading differences are much higher than the suggested threshold of 0.1. In all cases the HTMT values are below the threshold of 0.85. These results indicators that the discriminant validity is present in this study. The statistical values of each goodness of fit index met the threshold conditions, and the PLS-SEM had a good fitting effect on sample data, and the model passed the robustness test.

**TABLE 4:** Discriminant validity–Fornell–Larcker criterion and Heterotrait–Monotrait ratio.

Variables	Mean	S.D	1	2	3	4
1. Individual power (IP)	4.69	1.12	**0.813**	0.786	0.595	0.733
2. Regional power (RP)	4.964	1.22	0.672**	**0.774**	0.619	0.695
3. Cultural power (CP)	5.012	1.20	0.510**	0.510**	**0.751**	0.567
4. Guaranteed behavioral response (GBR)	5.06	1.05	0.657**	0.601**	0.486**	**0.903**

Significant level: *p* < 0.10; *p < 0.05; **p < 0.01; ***p < 0.001, Bold diagonal entries are square root of AVEs, Heterotrait–Monotrait ratios (HTMT) (Underlined) are below 0.85.

### Structural model assessment

This study followed Hair et al. (2012) to estimate the structural model. First, the results show minimal collinearity in the structural model as all VIF values are far below the common cutoff threshold of 5 Hair et al. (2012). Second, following the rules of thumb, the *R*^2^ values of GBR (0.493) exceed the minimum value of 0.10 recommended by Hair which is a satisfactory level of predictability as shown in Table 5. Similarly, results from blindfolding with an omission distance of 7 yield *Q*^2^ values well above zero (Table 5). This supporting the model’s predictive relevance in terms of out-of-sample prediction. Further analysis of the composite-based standardized root mean square residual (SRMR) yields a value of 0.063, which conenterprises the overall fit of PLS path model (Henseler et al., 2014). Applying the bootstrapping procedure (5,000 bootstrap samples; no sign changes) provides the *p*-values as well as the corresponding 95% bias-corrected and accelerated (BCa) bootstrap confidence intervals (Table 5). The empirical results support the vast majority of hypothesized path model relationships among the constructs.

**TABLE 5:** Significant testing results of the structural model path coefficients.

	Path coefficient	*t*-value	*P*-value	95% BCa confidence interval	Conclusion
Individual power (IP) → Guaranteed behavioral response (GBR)	0.416	6.993	0.000	(0.294, 0.529)	H1 supported
Regional power (RP) → Guaranteed behavioral response (GBR)	0.246	2.937	0.003	(0.066, 0.392)	H2 supported
Cultural power (CP) → Guaranteed behavioral response (GBR)	0.149	2.988	0.003	(0.058, 0.253)	H3 supported

SRMR composite model = 0.063.

R^2^_GBR_ = 0.493; Q^2^_ICMS_ = 0.391.

Based on the above results, the analysis is as follows:

First, the standardized path coefficients of IP, RP, and CP to GBR were significant at 0.001, 0.05, and 0.05 levels, respectively. Theoretical hypotheses H1, H2, and H3 were all effectively verified, indicating that the cognitive basis of farmers’ choice of security mode conforms to the distributed cognitive framework, and farmers’ choice behavior of security mode is influenced by three cognitive levels: IP, RP, and CP.
Second, the standardized path coefficient of RP → GBR is 0.246. RP is the main cognitive level that affects farmers’ security behavior response. Factor loading coefficients of RP1, RP2, RP3, RP4, and RP5 of RP observation indexes were 0.763, 0.812, 0.749, 0.783, and 0.762, respectively. It shows that the increase of household income can effectively promote the response of peasant households’ security behavior, and the area proportion of land transfer and their yearning for a better future life, while the happiness of peasant households’ life and the re-employment ability of family members also affect peasant households’ security behavior to a certain extent.
Thirdly, the standardized path coefficient of CP → GBR was 0.586, which was relatively small among the three cognitive levels of distributed cognition, indicating that CP was an important cognitive level affecting farmers’ security behavior. The factor loading coefficients of CP1, CP2, CP3, CP4, and CP5 were 0.745, 0.771, 0.761, 0.770, and 0.707, respectively. It shows that farmers’ satisfaction with endowment insurance policy has a significant impact on their security behavior, followed by their satisfaction with agricultural subsidies and medical insurance policy, and the promotion effect of policy publicity on farmers’ security behavior needs to be improved.
Fourthly, the standardized path coefficient of IP → GBR is 0.416, which is largest among the three cognitive levels of distributed cognition, indicating that IP is an effective cognitive level affecting farmers’ security behavior. Factor load coefficients of IP1, IP2, IP3, IP4, and IP5 are −0.776, 0.844, 0.846, 0.788, and 0.811, respectively, indicating that with the improvement of education level, farmers are more inclined to take appropriate security behaviors. The higher the level of non-agricultural livelihood of farmers, the higher the corresponding degree of their security behavior will be. It should be noted that the effect of gender and age on IP is negative, indicating that for land-lost farmers, female group and elderly farmers have certain limitations on their cognition of security behavior.
Fifthly, the factor loads of GBR1, GBR2, and GBR3 on GBR are 0.860, 0.934, and 0.912, respectively, and reach the significance level of 0.01, indicating that among the three security modes, land-lost farmers are more inclined to choose policy-based security, followed by traditional savings security, and their acceptance of market-based security needs to be improved.

## Conclusion and policy recommendations

### Conclusion

First, landless farmers’ choices of guaranteed methods follow the basic framework of distributed cognition, and their behavioral decision-making mechanisms are influenced by a combination of cognitive levels such as individual power, territorial power, and cultural power. Among them, regional power is the main cognitive level, cultural power is an important cognitive level, and personal power is an effective cognitive level.

Second, the increase of household income can significantly improve the level of farmers’ guaranteed behavior, and promoting farmers’ income increase is the core of improving the level of rural social guaranteed; the function of land guaranteed can be replaced by other guaranteed methods to a certain extent, and farmers have the potential incentive to withdraw from land and choose other guaranteed methods; the atmosphere of farmers’ family life and household labor endowment can promote their guaranteed behavior to a certain extent.

Third, farmers’ guaranteed behavior is largely influenced by the policy, and the performance evaluation of policy implementation (satisfaction) has a greater effect on farmers’ guaranteed behavior than policy publicity, suggesting that landless farmers’ behavioral decisions are more likely to be performance-oriented than opinion-oriented, and the effect of policy implementation promotes farmers’ adverse choice of guaranteed methods.

Fourth, the improvement of farmers’ individual quality (including literacy and part-time work ability) can promote their guaranteed behavior to a certain extent, but the “accumulation of experience” as they grow older may inhibit them from taking effective guaranteed measures. In addition, the survey found that there is gender discrimination in the decision-making process of landless farmers’ households, and the guaranteed needs of female groups are difficult to be met effectively.

Fifth, there is a clear preference in the choice of protection methods among landless farmers, with government-led policy-based protection being the mainstay of the rural social guaranteed system, while farmers still rely to some extent on traditional savings methods, and the acceptance of market-based insurance products and financial services in rural areas still needs to be further enhanced.

### Policy recommendations

First, regulate land transfer and improve the level of protection. With the agglomeration effect of urban development becoming more and more prominent, a large number of rural laborers are moving to the cities, the rural areas in the suburbs tend to decline, and the abandonment and abandonment of arable land are serious, and the small farmer economy is in trouble. In this context, China has tried to solve the real problems of abandonment and fragmentation of arable land by implementing land management rights transfer. Scholars have pointed out that the standardized implementation of land transfer policy can effectively improve farmers’ welfare and promote farmers’ household income, but at the present stage, land transfer in China still suffers from the “double-low dilemma” of low level and low efficiency (Ma et al., 2020). The results of the analysis of farmers’ perception of “territorial power” show that landless farmers have a tendency to “exchange land for guaranteed,” and the transformation of family livelihood through land transfer can effectively promote the response of farmers’ guaranteed behavior. Based on this, the government should accelerate the establishment of a rural land property rights trading platform, standardize the flow process, expand the scope of the flow, and introduce a market-based price competition mechanism to ensure the reasonable realization of farmers’ land property rights, so as to further improve the quality and level of farmers’ guaranteed behavior.

Second, strengthen policy guidance and establish a feedback mechanism. China is at a critical stage of transition from traditional smallholder economy to agricultural modernization, and the livelihood environment of rural households is subject to exogenous shocks from institutional changes, making them typically “risk averse” (Wilson et al., 2018). Studies show that government subsidies, insurance and financial policies through fiscal transfers are still the main supply of social guaranteed services in rural areas, and rural residents are largely path-dependent on them. As the demand side of risk protection, the farming community has the initiative and necessity to supervise government actions. However, the current rural social guaranteed system has not yet established a reliable policy feedback mechanism. Government departments focus unilaterally on the “top-down” system design, ignoring the possible intersection of various risk factors and uncertainties, and the lack of “bottom-up” complaint channels for farmers’ demands for protection. The analysis of farmers’ perceptions of “cultural power” shows that farmers’ subjective evaluations of the effectiveness of policy implementation have a more significant impact on their response to protection behavior than the government’s policy promotion efforts. If this cognitive “anchoring effect” persists for a long time, it will definitely have a negative impact on the government’s credibility. On the one hand, the government should further strengthen its policy propaganda work and improve the efficiency of policy guidance and policy implementation; on the other hand, relevant departments should establish a feedback mechanism for grassroots farmers on various protection policies and transform farmers’ subjective evaluations into objective evaluations of policy performance, so as to ensure the realism and effectiveness of various protection policies.

Third, be vigilant about the fragmentation of farm households and focus on vulnerable groups. With the continuous refinement of social division of labor, many aspects of agricultural production have been replaced by specialized social service institutions, and the group of farmers has transformed from traditional “agricultural production labor” to new “agricultural business decision makers.” The survey found that the typical rural areas in the sample regions are now in a state of coexistence of multiple agricultural business entities, with pure farmers, semi-part-time farmers and non-farmers all accounting for a certain proportion, and the farming groups showing a heterogeneous and differentiated development. The results of the analysis of farmers’ perception of “personal manpower” show that the higher the level of education and part-time employment of farmers, the higher the level of their guaranteed needs; as they grow older, their motivation to adopt effective guaranteed behavior decreases significantly; at the same time, female farmers recognize guaranteed behavior less than male farmers. The above analysis shows that the divergence of farmers’ guaranteed behavior mainly comes from the difference of education and part-time employment. The government should be alert to the possible class division and class conflict of farmers caused by this divergence trend, especially for the disadvantaged groups of farmers (such as women and the elderly), and should give some attention and policy favor to them, so as to maintain social equity and justice.

## Data availability statement

The original contributions presented in this study are included in the article/supplementary material, further inquiries can be directed to the corresponding author.

## Ethics statement

Ethical review and approval was not required for the study on human participants in accordance with the local legislation and institutional requirements. Written informed consent from the patients/participants or patients/participants legal guardian/next of kin was not required to participate in this study in accordance with the national legislation and the institutional requirements.

## Author contributions

HD and HW: methodology and software. HD and YL: formal analysis, resources, data curation, and writing—review and editing. HD: investigation. YL: writing—original draft preparation. HW: supervision and project administration. All authors have read and agreed to the published version of the manuscript.
